# Out of sight but still in mind: Developing an expectation for surprises by formalizing unknowledge in a contemporary risk‐assessment framework

**DOI:** 10.1111/risa.17661

**Published:** 2024-10-08

**Authors:** James Derbyshire, Terje Aven

**Affiliations:** ^1^ Chester Business School University of Chester Chester UK; ^2^ Department of Safety, Economics and Planning University of Stavanger Stavanger Norway

**Keywords:** probability, surprise, uncertainty, unknowledge

## Abstract

Extreme events like the credit crunch, the September 11th attacks, the coronavirus pandemic, and Hamas’ attack on Israel each have in common that they should *not* have come as a surprise, yet still did. One reason surprises happen is that a risk assessment reflects the knowledge of the assessors, yet risk also includes uncertainties that extend beyond this knowledge. A risk assessment is thus susceptible to surprises as it focuses attention on what is known. Developing an expectation for surprises is key to their avoidance and requires that risk assessors specifically consider their “unknowledge”—that is, what they do *not* presently know about an event, outcome, or activity and its potential consequences and triggers. One way to emphasize the need for risk assessors to consider unknowledge is to explicitly include it as a separate component in risk‐assessment frameworks. This article formalizes the inclusion of unknowledge in a contemporary risk‐assessment framework.

## 1 INTRODUCTION

Traditionally, risk assessments employed probabilities to express uncertainties. However, contemporary risk‐assessment frameworks recognize the need to “go beyond probability” (Aven, 2016) by including a component that reflects a judgment on the strength of knowledge^1^ lying behind a measure of uncertainty, such as a probability. The inclusion of this component recognizes that using probabilities to represent uncertainty without also considering the strength of knowledge on which they depend could mislead. For example, a highly consequential event could be assigned a very low probability, but if the knowledge on which its probability depends is scant it would be inadvisable to disregard the event based solely on its low probability.

Only by considering relevant knowledge that is *not* presently available alongside that which is, can the strength of knowledge be properly assessed. Assessing the strength of knowledge on which an uncertainty measure (e.g., a probability) depends *implicitly* recognizes that uncertainty can arise from an absence of knowledge—that is, what Shackle (1955, 1961) called “unknowledge” and Keynes (1936) called “ignorance,” with this article employing the former term throughout. Moreover, this source of uncertainty is explicitly recognized in the emphasis placed by contemporary risk‐assessment frameworks on concepts and tools for mitigating surprises, such as signals and early warnings, assumption deviation risk, systems thinking, and black‐swan theory (e.g., Turner & Pidgeon, 1997; Taleb, 2007; Weick & Sutcliffe, 2007; Paté‐Cornell, 2012; Aven, 2014; Bjerga & Aven, 2016).

Yet, despite the contemporary risk‐assessment framework's recognition of unknowledge and the potential for surprises it implies, governments and other organizations continue to be surprised by highly consequential events, such as the credit crunch, the September 11th attacks, the coronavirus pandemic, and most recently, Hamas’ attack on Israel. This suggests awareness of the importance of unknowledge and the potential for surprises it implies remains underdeveloped, at least among the consumers of risk assessments if not necessarily their producers. Given these continued surprises, to emphasize the attention it warrants, we suggest that unknowledge be formalized as a specific component in risk‐assessment frameworks. This formalization might be especially helpful in the age of “evidence‐based policymaking” (Pawson et al., 2011), which focuses attention on what is known, thereby directing it away from what is unknown and in so doing increasing the potential for surprises (Derbyshire, 2020).

Unknowledge also warrants explicit inclusion as a separate component in risk‐assessment frameworks because, in its most intractable guises, it is more than simply a matter of incomplete information, which is what it is when evidence is scattered between different individuals and organizations that fail to transform it into knowledge of a risk by bringing it together and “connecting the dots,” leading to a surprise (Bazerman & Watkins, 2005; Glette‐Iversen & Aven, 2021). That is, conceptually, its most tractable form, in which all the relevant evidence (whether available or unavailable to any one individual entity such as a particular organization) already resides on and is part of the knowledge landscape.^2^ Under such circumstances, the aim is to connect the dots through intersubjectivity^3^ and in so doing gain a more‐or‐less complete picture of this landscape's terrain, allowing for gaps in knowledge to be filled in through inference based on what is known.

In its less tractable form, the concept of unknowledge recognizes the tendency for a major development or a crucial decision^4^ to change radically both the knowledge landscape and the space of possibilities it implies (Shackle, 1955, 1961). Remedying this form of unknowledge is not simply a matter of “connecting the dots” through intersubjectivity, and then filling in any remaining gaps in knowledge through inference. The known elements are too few, and the gaps in knowledge are too numerous and too wide to be filled through inference. The unknowns are not merely unknown to any one entity but to all, and in some cases take the form of unknown unknowns.

Unknowledge, therefore, has a dynamic aspect in interaction with knowledge. Together, the elements of both form the terrain of a constantly evolving knowledge landscape. The dynamism of this landscape and the tendency for its terrain to be radically changed by a major development or crucial decision requires that the changing relative extent and importance of its known and unknown elements be explicitly considered in a risk assessment. As such, this article does two things:

1. It argues for and elaborates on the need for explicit consideration of unknowledge and its dynamism within a comprehensive risk assessment.
2. It formalizes the inclusion of unknowledge and its dynamism as separate components in a contemporary risk‐assessment framework.

This article is complemented by others published separately, which summarize methods for assessing the extent and importance of unknowledge and demonstrate the feasibility of the approach described in this article. The focus of this article is on formalizing the inclusion of unknowledge in a contemporary risk‐assessment framework.

## 2 THE EPISTEMIC COMPONENT IN CONTEMPORARY RISK‐ASSESSMENT FRAMEWORKS

O'Driscoll and Rizzo (1996, p. 64) long ago stated that risk analysis is “essentially a weighting of possibilities already known.” The implication was that it fails to take account of the “genuine uncertainty” arising from the “unpredictable growth of these possibilities” over time, which leads to the emergence of knowledge “gaps” (O'Driscoll & Rizzo, 1996, pp. 64–65). Whether this statement was ever true is debatable: even traditional risk analysis recognized this problem by including techniques like sensitivity analysis. Regardless, the debate is anyway redundant because of the development in recent decades of new risk‐assessment frameworks that “go beyond probability” (Rosa, 1998; Renn, 2008; Aven, 2012). These frameworks seek to account for uncertainty in the very broadest sense of the term. This development is very welcome and is manifest most explicitly in contemporary risk‐assessment frameworks’ inclusion of a component representing the set of knowledge underpinning a measure of uncertainty such as a probability.

Consider, for example, the (C, U) risk‐assessment framework (Aven et al., 2011; Aven, 2013, 2016, 2020; Flage et al., 2014) in which risk is understood very broadly as the two‐dimensional combination in (1):

(1) $$\left(C,U\right)$$

where C is the consequences of the activity considered (including not only its outcomes but also its initiating events) and U is the associated uncertainty (i.e., what will C be?). From (1), risk is characterized by the risk assessment using three components, as in (2).

(2) $$\left(C\prime ,Q,K\right)$$

where C′ is a set of specified consequences (e.g., the number of fatalities), Q is an appropriate measure or description of uncertainty, and K is the knowledge on which C′ and Q rely. Under this framework, properly accounting for uncertainty requires all three components. While under this framework, a subjective probability may be assigned as the measure of uncertainty (i.e., Q) for any event or outcome whatsoever, the framework recognizes that it could be largely meaningless and may even be misleading if not assigned alongside consideration of the strength of knowledge on which it depends. The framework thus emphasizes the need to always complement the use of a subjective probability with a judgment about the strength of knowledge on which it is based (Aven, 2016).

Based on this framework, a risk assessment therefore reflects the knowledge of the assessors making it. However, as the framework implicitly recognizes, risk also includes uncertainties that extend beyond this knowledge, taking the form of what are presently unknowns, such as “systemic risks” from second‐order effects on interacted systems (Renn et al., 2022), which may give rise to surprises. These are implicitly recognized in the gap between C′ in (2), which represents the set of consequences presently known or conceivable to the assessors, and C in (1), which represents the actual consequences that could occur regardless of whether they are known or conceivable to the assessors. Hence, the emphasis placed by those setting out this framework is on concepts and tools for dealing with unknowns and surprises, such as signals and early warnings, assumption deviation risk, systems thinking, and black‐swan theory (e.g., Turner & Pidgeon, 1997; Taleb, 2007; Weick & Sutcliffe, 2007; Paté‐Cornell, 2012; Aven, 2014; Bjerga & Aven, 2016).

Critically, while taking account of uncertainty in its broadest form by including a component focused on knowledge and its strength should increase confidence in a risk assessment and its comprehensiveness, care is needed to ensure the assessment does *not* imbue its users with an unwarranted confidence that all uncertainties have been captured by it. A risk assessment *must* make its users cognizant of the potential for surprises—that is, that the beliefs and assumptions comprising the set of knowledge (K) on which it is based could be wrong or could be incomplete in some important way. Only by so doing might it stimulate a willingness to develop contingencies or to take appropriate precautionary decisions. Overconfidence in the risk assessment's conclusions and the quality of the knowledge on which it is based will act against this willingness, where the key aspect of quality here concerns the potential difference between the judgments made about consequences (C′) and the actual consequences (C). Given their emphasis on concepts and tools for mitigating susceptibility to surprises, the developers of this framework are unlikely victims of overconfidence in the results of a risk assessment that employs it. But that may be less true of someone commissioning or using its output, who may be less well‐versed in the modern and comprehensive understanding of risk, which includes uncertainty in the broadest sense of the term. There are two reasons why the framework may fail to temper the latter's overconfidence in the risk assessment's results, despite the efforts made by the framework's originators and developers to emphasize the need to consider unknowns and surprises.

First, there is the emphasis placed today on “evidence‐based policymaking,” which puts empirically derived knowledge on a gold‐standard pedestal and which in so doing relegates the more abstract concept of unknowledge to a secondary consideration (Pawson et al., 2011). This is not a problem with the framework per se, but the culture in which it may be applied. Nevertheless, it is a problem that the framework must overcome to have its intended effect of accurately communicating risk in its broadest sense and especially to policymakers. Second, the human tendency to focus on what is known and to overlook what is unknown (e.g., confirmation bias), which may be exacerbated by the framework's otherwise very welcome inclusion of a component representing knowledge (i.e., K). The potential is for the inclusion of this component to have the unfortunate by‐product of unduly focusing the risk assessment on what is known, causing the need to deploy the complementary concepts and tools for assessing and dealing with unknowns and surprises to go unrecognized.

This is problematic because, when it comes to crucial decisions involving risk broadly conceived to include uncertainty of all types, there is sometimes more that we do *not* know than we do know (Rowe, 1994). It is therefore imperative to consider what we do *not* know alongside that which we do (Rowe, 1994) as surprises would tend to arise from what is presently unknown. The adjustments to the framework set out in this article seek to refocus it in that way—or, perhaps more accurately, to rebalance it to ensure it stimulates equal consideration of unknowledge alongside knowledge.

In the next section, we show why unknowledge demands recognition as a distinct and separate component within the (C, U) framework. The discussion therein not only illustrates the dangers of failing to consider unknowledge specifically but also illustrates how unknowledge has a dynamic aspect in interaction with knowledge, requiring the changing relative extent and importance of both over time (and the effect of these changes on C′ and Q) to be explicitly considered in a risk assessment. As we then show in the final section, this explicit consideration is enabled by including in the framework a distinctive and separate term for unknowledge.

## 3 THE DISTINCTIVENESS OF UNKNOWLEDGE

The September 11th, 2001, terrorist attack on the USA has been referred to as a “black swan” because it came as a surprise (Glette‐Iversen & Aven, 2021). Yet, analyses have shown that some government agencies knew of Islamic terrorist groups’ plans for an attack involving the hijacking of aircraft (Glette‐Iversen & Aven, 2021). The attack was a surprise because there was a failure to “connect the dots” (Glette‐Iversen & Aven, 2021, p. 10). What was unknown in a more fundamental sense were details about the specific targets and scope of the attack. Yet, these unknowns could have been inferred from what *was* known. For example, the World Trade Centre was a prominent building in the USA's most high‐profile city and had been the subject of terrorist attacks previously. It did not require a huge leap of the imagination to foresee it as a potential target. If government agencies had “connected the dots” in terms of what they *did* know, they could have filled in the gaps related to what they did not. However, care must be shown when making such evaluations with hindsight. At the point of analysis, the space of scenarios and events is wider, and the uncertainties are larger.

As this example illustrates, what an individual does not know is at least to some extent subjective to them. To draw an analogy, black swans were no surprise to aboriginal people in Australia, who had known about them for thousands of years (Paté‐Cornell, 2012). For Aborigines, white swans would have been surprising. The September 11th attack is an example of a surprise in which many of the relevant knowledge elements were unknown knowns in that they were known by some but were not known to all the parties tasked with making a risk assessment. The failure was therefore in terms of an effective knowledge management and aggregation system (Paltrinieri et al., 2012). It was one of failing to connect the dispersed knowledge elements so that what *was* known could be used to make inferences about what was *not* known: exact location, specific targets, and scope, etc. Granted, filling these gaps would not have been a trivial matter as there were many possibilities to consider, but the problem was nevertheless one amenable to analysis. It was a matter of applying appropriate resources to this task of analysis, ensuring that dispersed knowledge elements were fully diffused to all relevant parties such that they could be aggregated, and then narrowing the potential possibilities by filling in the remaining gaps, leading to recognition of the need for urgent action.

A more difficult‐to‐grapple form of unknowledge stems from the tendency for major developments and “crucial decisions” (Shackle, 1955, 1961) to change radically both the knowledge landscape and the space of possibilities it implies (Shackle, 1955, 1961). This is not a matter of connecting the dots and then making inferences that fill in the remaining gaps. A major development or crucial decision leads to the obsoleting and deleting of some present knowledge elements (both knowns and unknowns), the radical altering of other knowledge elements, and the creation of new knowledge elements in the form of new knowns and unknowns. The terrain of the knowledge landscape is immediately, radically, and irrevocably reconfigured by a major development or crucial decision, meaning large parts of the new landscape then formed are unknown in a fundamental way, which in turn radically revises the range of possibilities and their potential consequences and impacts as well as their associated uncertainty.

The decision of the UK government *not* immediately to implement a strict quarantine in the face of the coronavirus pandemic in February 2020 was an example of just such a crucial decision. Following the decision, new unknown knowledge elements emerged as associated with new possibilities: for example, whether the virus would spread throughout the country leading to fatalities in the millions, whether the National Health Service would be overwhelmed or whether a type of herd immunity might develop. In bringing these new unknown knowledge elements into being, the strength of knowledge on which judgments about the possibilities for and consequences of the pandemic's spread depended was radically altered by the decision, as reflected in the then changed extent and importance of the known and unknown knowledge elements comprising the radically remade knowledge landscape.

**FIGURE 1:**
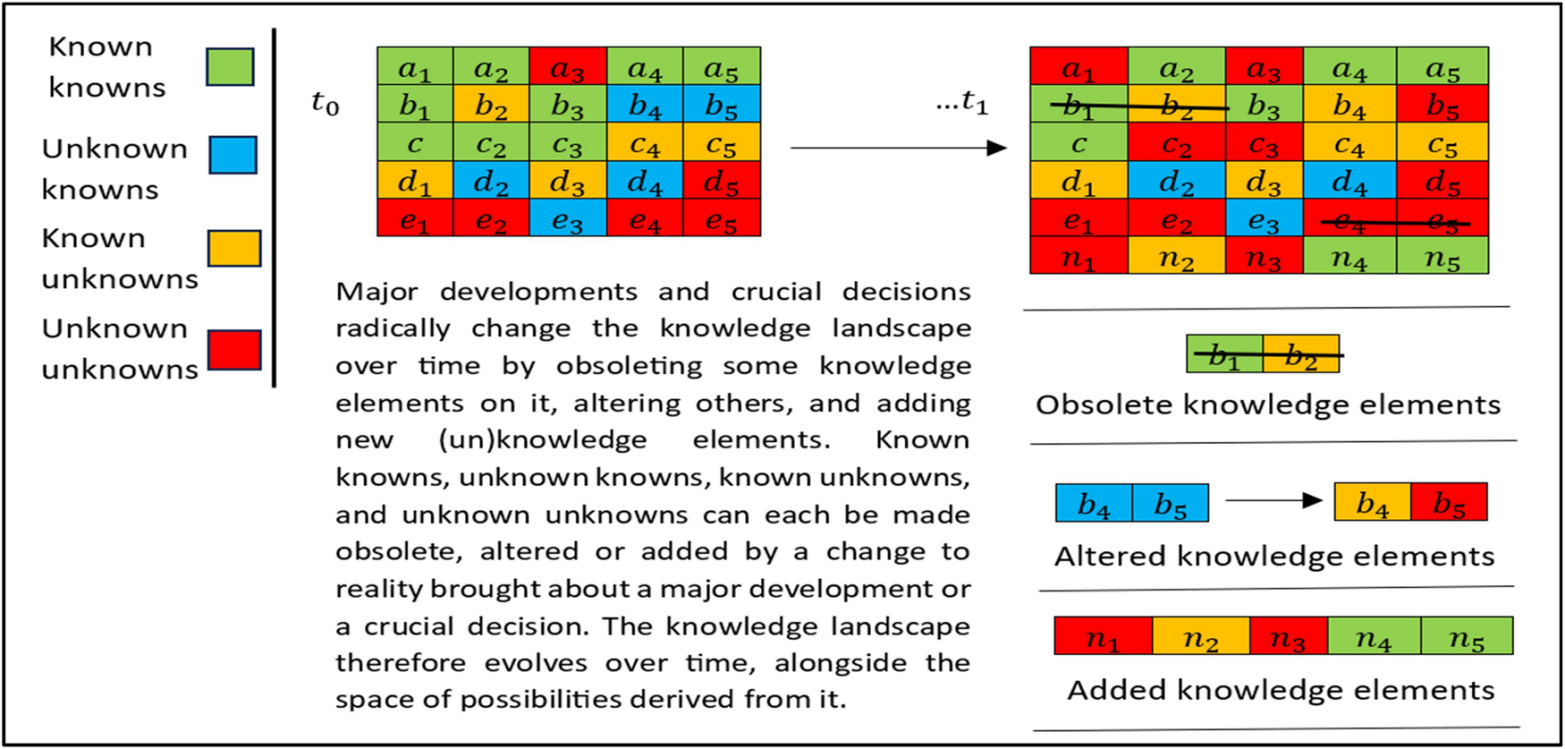
Major developments and crucial decisions radically change the knowledge landscape.

This dynamic process is illustrated in Figure 1, in which a knowledge landscape is composed of known and unknown knowledge elements based on the categories famously described by Donald Rumsfeld. Drawing on Paltrinieri et al. (2012) and Feduzi et al. (2022) and expanding on Rumsfeld, 2002a, 2002b, 2011), the categories of known and unknown knowledge elements are

- *known knowns*: elements of knowledge of importance to assessing the focal risk that are well established and fully known to those tasked with assessing it;
- *known unknowns*: elements of knowledge that the risk assessors know to be of importance to assessing the focal risk, but which are incomplete or entirely missing, or about which there is currently conflicting evidence^5^;
- *unknown knowns*: elements of knowledge relevant to assessing the focal risk that are known to someone or documented somewhere—that is, by an individual, government department, or other body―but which are presently unknown to those tasked with making the risk assessment;
- *unknown unknowns*: elements of knowledge that would be of importance to assessing the focal risk if they were known but which are presently unknown to anyone including those tasked with making the risk assessment, who, furthermore, lack awareness of not knowing them.

As noted by Derbyshire et al. (2023), this last category may seem unamenable to assessment. On the face of it, one cannot assess what one does not know if one is unaware of not knowing it. Yet, there is some evidence of an ability to assess the extent to which a focal event, outcome, or activity may be subject to unknown factors of importance that may only be revealed over time (Loch et al., 2008; Svetlova, 2021; Derbyshire et al., 2023). As noted previously, this article is complemented by others, published separately, which summarize methods for assessing the extent and importance of unknowledge, and which set out new methods for engaging in structured acts of imagination that can aid assessment of unknown unknowns by transforming them into known unknowns.

Figure 1 illustrates how the changes wrought by a major development or crucial decision affect the knowledge (K) on which the assessment of consequences (C′) and uncertainty (Q) depend by radically changing the knowledge landscape. This makes a risk characterization a moving target requiring anticipation of how changes wrought by a new policy, decision, or regulation alter the knowledge landscape, the range of consequences and impacts implied by it, and their uncertainty.

Vickery et al. (2022) describe the problem of making evidence‐informed decisions during the pandemic and the many situations of incomplete, evolving, and/or conflicting evidence (which are all types of unknowledge) to which it gave rise. The knowledge landscape was in turmoil as each new development and crucial decision radically altered the extent and importance of its known and unknown elements. This in turn affected the range of the consequences and impacts (C′), and their uncertainty descriptions (Q), which are derived from the knowledge landscape. Pandemic decision‐making was thus fraught with uncertainty and dynamism. While a pandemic might seem like an unusually extreme circumstance in this regard, Pawson et al. (2011) show this to be the standard predicament faced by evidence‐based policymaking, even in more mundane times.

What we might call “knowledge turbulence” of this type can occur at any time because of invention, discovery, and innovation, which may radically change the knowledge landscape in the illustrated way. As the economist of uncertainty G. L. S. Shackle puts it (Shackle, 1966, p. 757):

New knowledge is destructive…it overthrows more or less radically and completely the structure of ideas we have hitherto relied on. It throws light from new, unsuspected directions, it makes everything look different, it upsets all calculations, it states new axioms, it leaves nothing as it was.

The pandemic again provides an excellent example in the invention of vaccines against coronavirus, which radically altered the known and unknown possibilities from that moment of the pandemic onwards. For example, it did this by introducing new unknown knowledge elements related to whether a sufficiently large proportion of the population would willingly be inoculated, whether a vaccine would have the broad efficacy its rapid invention and testing implied and whether it would have side effects or complications.

We might also think about this knowledge turbulence in relation to artificial intelligence (AI), which currently has unknown, and perhaps presently unknowable, potential consequences. There will be risk assessors somewhere tasked with assessing its risks, but the knowledge landscape on which they make these assessments is currently in turmoil. Each new milestone in AI's development may raise more questions than it answers, each with the potential to radically change the knowledge landscape by altering the territory occupied by the known and unknown elements of which it is composed, with implications in turn for the potential consequences and their uncertainties, which are derived from it.

Immanuel Kant established “the principle of question propagation,” which implies that every answer science provides simply begets another question. George Bernard Shaw similarly stated that “Science never solves a problem without creating ten more.” As evident in pandemic decision‐making, among the primary changes to the knowledge landscape induced by an invention or discovery (e.g., a vaccine), or by a crucial decision (e.g., to “lockdown”), or by a new policy or regulation (e.g., social distancing), are those related to human behavioral responses. The reflexive human response to a major change or crucial decision can be highly uncertain.

For example, if the Bank of England published a risk assessment suggesting they had found strong evidence that a new credit crunch may occur soon and had assigned this possibility a high probability based on this strength of knowledge, it is unclear whether this would prevent or precipitate the occurrence of a new credit crunch (Thompson, 2022). It may stimulate an immediate regulatory response which negates the anticipation, or it might stimulate a run on the banks, which would fulfill it. Human reflexivity of this type (i.e., the uncertainty associated with how people may respond) can be exceedingly difficult to anticipate, thus greatly expanding that part of the knowledge landscape occupied by unknowledge (Derbyshire, 2020).

These factors make unknowledge something more than just an individual's or specific entity's (e.g., a government department's) incomplete set of knowledge, as in the case of the September 11th attacks on the USA. As we have noted, while certainly not a trivial or simple endeavor, and while easier to say in hindsight than to do with foresight, that case could nevertheless have been resolved by “connecting the dots” of dispersed knowledge through intersubjectivity (see footnote 3). Rather, the problem here is ontological: a fundamental change to reality brought about by a major development or crucial decision. Following this logic, it is not sufficient for a risk assessment merely to consider the strength of knowledge in terms of what is presently known and unknown about a focal risk. Much more problematically, it must also consider how the known and unknown knowledge elements might change over time, both individually and in relation to each other. Specifically, it should consider how they might be changed by a decision or the absence of a decision, a new regulation, or some other major development, which takes place over time. In the next section, we consider how the (C, U) framework can be extended to stimulate such consideration by including unknowledge and its dynamism as specific and separate components.

## 4 FORMALIZING UNKNOWLEDGE AS A DISTINCTIVE AND SEPARATE COMPONENT IN THE (C, U) FRAMEWORK

We argue that it is necessary to emphasize the importance of unknowledge and its dynamism within a comprehensive risk assessment by expanding the characterization of risk in (2) to include the new components set out in (3).

(3) $$\left(C\prime ,Q,K,\mathrm{UK},T\right).$$

The component representing knowledge (K), which previously combined both knowledge and unknowledge, now focuses only on knowledge that *is* available and relevant to the risk assessment. Note that the judgment of the strength of knowledge is an aspect of Q. The components now added (UK and T), respectively, represent the current absence of knowledge that would be relevant to the risk assessment if it were available (UK) and the change in both knowledge (K) and unknowledge (UK)―and, concomitantly, C′ and Q―over time (T), which may be brought about by anticipated developments, including the making of a decision or the implementation of a new regulation. In this way, the need to consider both knowledge *and* unknowledge and perhaps (depending on the nature of the focal risk) even to focus on and emphasize unknowledge, is made explicit. The set of knowledge K might include a key assumption and uncertainty about its correctness (reasonability) would become one of the elements considered when assessing the strength of knowledge (Q). The UK component adds another dimension, namely that these judgments could be inaccurate or wrong, or that the set of knowledge K is missing something important, which would affect the risk assessment in one way or another if it were not missing.

The risk characterization set out in (3) can account better for the effect of crucial decisions than can that in (2) because it places emphasis on how the relationship between the knowledge components (K and UK) changes over time (T). Reconsider the decision of the United Kingdom's government *not* immediately to implement a strict quarantine in the face of coronavirus in February 2020. According to Adam (2020), this initial decision was influenced to a degree by what was perceived to be knowledge of the proportion of those admitted to the hospital who would need ICU treatment (Derbyshire, 2022), which was in part based on data from China. Yet, the knowledge related to this matter (i.e., the proportion of patients requiring ICU treatment) was weak because of much important unknowledge (UK), such as whether the proportion of patients requiring ICU treatment might be higher in the United Kingdom due to presently unknown factors, whether the virus that had reached the United Kingdom was of the same strain or one with greater virulence, and whether the proportion of patients requiring ICU treatment might change over time as the pandemic progressed meaning the initial estimate was an underestimate. In fact, data that then came from Italy did indeed suggest it was an underestimate (Adam, 2020).

In other words, knowledge was therefore, at this very early stage of the pandemic, greatly overshadowed by unknowledge. Yet, the decision not to implement a strict quarantine immediately was influenced by what *was* known, and according to Adam (2020), this knowledge was deployed in the form of an initially dubious model parameter. Herein, we see the danger of overly focusing on knowledge, as might *inadvertently* be brought about by (2), and the need explicitly to take into consideration unknowledge, as in (3). An explicit consideration of what was unknown might have stimulated a very different, precautionary type of a decision, leading to an immediate and strict “lockdown.”

We emphasize once more that consideration of unknowledge is already an *implicit* part of the (C, U) framework in (1) and its associated risk characterization in (2). But for the reasons outlined, consideration of unknowledge should become an *explicit* and integral part of a comprehensive risk assessment, as in (3). The risk characterization provided by (2) could miss important aspects of unknowledge: important influencing events and factors might be ignored, and beliefs and assumptions supporting the uncertainty description and the assessment of the strength of knowledge could be wrong.

**FIGURE 2:**
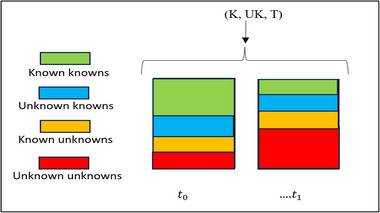
Assessing changes to the knowledge landscape over time (adapted from Derbyshire et al., 2023).

The dynamic assessment enabled by (3) is illustrated in Figure 2, which shows how the components K and UK can be considered in unison. A change to reality brought by a decision, new regulation, invention, or by some other means, changes the relative extent of knowledge and unknowledge. Changes to the knowledge landscape that take place over time as illustrated in Figure 1 can be assessed by considering the change they bring to the relative extent of aggregated known and unknown knowledge elements as shown in Figure 2. Since we are talking here about the somewhat abstract matter of the obsoleting, altering, or creation of knowledge and unknowledge over time, the matter will rarely be an exact one amenable to calculation. It will always be a matter of judgment.

As noted, in articles published separately, we later set out new methods for engaging in structured acts of imagination, which enable the considerations needed to create the UK and T components shown in Figure 2. The present article's aim has been to illustrate why it is necessary to make such considerations an explicit and formal part of a risk assessment. As demonstrated, this helps to ensure that the (C, U) framework's otherwise welcome recognition of the need to underpin a risk assessment with consideration of the strength of knowledge on which it relies does not inadvertently result in unknowledge being overlooked.

## 5 CONCLUSION

The phrase “out of sight, out of mind” suggests that something that is not clearly visible may not only be invisible to the senses but also to the mind. As the phrase recognizes, the human tendency is to focus on what *is* there. Absences are more difficult to conceive, more abstract, and easier to overlook. Moreover, this tendency is further exacerbated by confirmation bias, which reinforces certitude about what is known, making consideration of what is not still more unlikely. Furthermore, today's emphasis on evidence‐based policymaking places empirical knowledge on a gold‐standard pedestal, and, as a by‐product, diminishes in importance the abstract thought processes and acts of imagination through which unknowledge might be considered. Considering these tendencies together, that we are repeatedly subject to highly consequential surprises is not itself surprising. The surprising thing is that we are not surprised more often.

Risk analysis has in the past been caricatured as a weighing of possibilities already known, the implication being that it fails to consider the tendency for gaps in knowledge to form and grow over time. Yet, modern risk‐assessment frameworks fully recognize that in all but trivial cases such gaps in knowledge will be ever present, including at the point at which a risk assessment is first made. However, while this is a welcome development, in acknowledging this by incorporating components dedicated to the knowledge on which the risk assessment depends and an evaluation of its strength, contemporary risk‐assessment frameworks run the risk of focusing attention unduly on what *is* presently known about an event, outcome or activity and its consequences and potential triggers, at the expense of what is not.

To develop an appropriate expectation for surprises in the minds of those commissioning and using them, a risk assessment needs to place equal or even greater emphasis on what is unknown. Highly consequential surprises continue to happen in part because the need to consider what is not presently known is not formalized and made explicit within contemporary risk‐assessment frameworks. Including unknowledge as a specific component in such frameworks helps to ensure that what is out of sight is not necessarily out of mind. Developing an appropriate expectation for surprises in the minds of policymakers assists them in taking appropriate precautionary decisions and diminishes any overconfidence they may have in the results of a risk assessment. A concomitant benefit is to help policymakers understand that a decision, as well as the absence of a decision, changes both the knowledge landscape and the associated space of possibilities derived from it, often in a way that is irrevocable.

## Footnotes

1 This article follows the understanding of “‘knowledge” set out in Aven and Flage (2023) in which it is a belief about a focal matter that is justified by a set of evidence, which may include data and information of all types, but which could also include evidence in the form of testing, argumentation, modeling, or simulation. The concept of “strength of knowledge” refers to the strength of the beliefs forming the knowledge and the supporting evidence (Aven, 2016; Aven & Flage, 2023). It reflects both the quality of the evidence and how well the beliefs are supported by the evidence. The completeness of the evidence is an aspect of the strength of knowledge concept. An assessment of completeness would include a judgement as to the importance of a piece of evidence. For example, there could be more pieces of relevant evidence available than unavailable, but if those unavailable have potentially major implications in terms of the potential consequences and uncertainty of a considered risk, then the knowledge derived from the set of evidence would be deemed weak in strength. It is, therefore, not simply a matter of the completeness of the set of relevant evidence, but also one of the importance attached by the assessor to the evidence that is available and unavailable.

2 In this article, we illustrate our argument throughout by employing the concept of a “knowledge landscape,” which we conceive of as a bounded space composed of the set of all evidence considered to be relevant to a focal matter (such as a risk to be assessed), whether presently available or unavailable. We refer to the distinct, individual pieces of evidence of which this space is composed as “knowledge elements” because they are pieces of evidence that have been recognized as being of importance to an assessor's belief in the possibility of occurrence and the severity of consequences of the considered event, outcome, or activity, whether presently known or unknown. Figure 1 provides an illustration.

3 By “connecting the dots through intersubjectivity,” we simply mean that representatives of, for example, distinct government bodies and institutions, or different departments and branches within a business organization, exchange the knowledge they have available to them in order that each of them then possesses—at least for the period thereafter in which the knowledge landscape remains unchanged—the same set of knowledge, which has been rendered more complete than any one of them previously possessed in isolation due to the exchange.

4 According to G. L. S. Shackle, who originated the concept, a “crucial decision” is one that changes the very circumstances in which the decision is made so that no future decision can ever be made in similar circumstances again (Derbyshire, 2017).

5 An example of a known unknown might be the consequences of governments’ decision to implement a mask‐wearing policy during the coronavirus pandemic. Prior to the policy's implementation, evidence about its effectiveness in curtailing the spread of a virus’ was only partially available (Vickery et al., 2022) as it had never been attempted on the scale (i.e., across the whole of society) or for the duration it was intended to implement it.
